# Standardized Surgical Management for Cystic Dilation of the Bile Ducts Based on Clinical and Pathological Studies: A Narrative Review

**DOI:** 10.1155/2020/3432786

**Published:** 2020-09-15

**Authors:** Hong-Tian Xia

**Affiliations:** Department of Hepatobiliary Surgery, First Medical Center of Chinese PLA General Hospital, Beijing 100853, China

## Abstract

The surgical method of complete/radical cyst excision plus Roux-en-Y hepaticojejunostomy remains the primary therapy and the only effective treatment for cystic dilation of the bile ducts (CDBDs). However, the incidence of long-term postoperative complications is still high, as is the reoperation rate, and the potential for postoperative malignant transformation still exists. In recent years, significant progress has been made in understanding the pathogenic mechanism and pathological changes of adult CDBDs. Based on which, the surgical procedures for CDBDs have been revised to further improve their effectiveness. The purpose of this review is to systematically summarize the latest concepts of the etiology and pathogenic mechanism and the pathological changes of adult CDBDs. Based on the findings of these clinical and pathological studies, a comprehensive theoretical system in the surgical treatment of CDBDs has been established, which corrects many previous theoretical misunderstandings. The specific surgical method for each type of CDBDs and the key technical notes are also described in detail. Using these principles, treatment outcomes for CDBDs can be significantly improved, and the current high complication rate, reoperation rate, and rate of postoperative malignant transformation can be reduced.

## 1. Introduction

Surgical treatment remains the primary therapy and the only effective treatment for cystic dilation of the bile ducts (CDBDs). Around the year 2000, the surgical method of complete/radical cyst excision plus Roux-en-Y hepaticojejunostomy was established [1] and subsequently has been recognized by the surgical community. However, since the etiology and pathogenic mechanism of CDBDs are still not fully understood, the incidence of long-term postoperative complications is still high, including the reoperation rate, and the potential for postoperative malignant transformation still exists. Moreover, the postoperative cancer rate is high in certain types of CDBDs (such as Todani type IVa) [2]. In some cases, the postoperative cancer rate is even higher than for those who do not receive surgical treatment [3, 4]. As such, there is a great deal of room for improvement in the treatment of CDBDs.

Our medical center has treated a great number of adult patients who need reoperation due to lack of proper bile duct flow following CDBD surgery. It emphasizes the importance of standardized surgical procedures. In recent years, significant progress has been made in understanding the pathogenic mechanism and pathological changes of adult CDBDs. Based on these advances in knowledge, the surgical procedures for CDBDs have been revised to further improve their effectiveness. For over 20 years, we have been committed to improving the long-term treatment outcomes of patients with CDBDs and have published a series of reports on the surgical approaches for CDBDs, as well as the importance of radical excision and proper bile duct flow [2, 5–11]. At present, there is no review of the pathogenic mechanisms and surgical managements for different types of CDBDs. This review will summarize the latest concepts of the pathogenic mechanism, pathological changes, and surgical management of CDBDs, as well as describe standardized surgical procedures.

## 2. A Narrative Review

### 2.1. Classification of CDBDs

**Figure 1:**
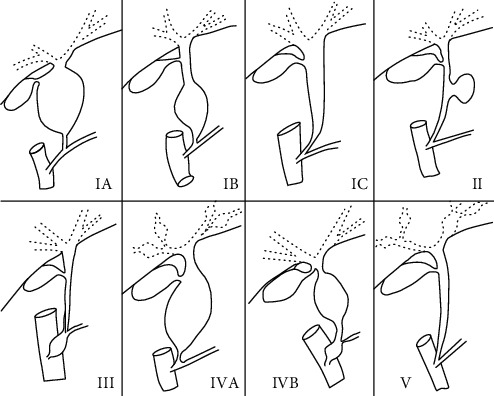
Todani classification of cystic dilation of the bile duct.

CDBDs is defined as an isolated or multifocal cystic dilation of an extrahepatic or/and intrahepatic biliary duct. The Todani classification system classifies CDBDs into 5 types according to the location of the biliary duct dilation and anatomical characteristics [3] (Figure 1), and this classification method is widely used to guide the choice of surgical procedure. Todani type I and type IV are the main types of dilatations, of which type I is the most common accounting for 50-80% of CDBDs [12]. Among type I CDBDs, type Ia is the most common, followed by type Ic. Type IV CDBDs account for 15-35% of all cases, of which type IVa is the most common type. Type II (2%) and type III (1.4-4.5%) CDBDs are both rare [12].

### 2.2. The Pathogenic Mechanism of CDBDs

The etiology of CDBDs is not fully understood, but several theories have been proposed. The most widely accepted theory is that there is an anomalous pancreaticobiliary junction (APBJ) [13]. An APBJ is defined as a junction between the pancreatic and bile ducts, which is located outside the duodenal wall and the control range of Oddi's sphincter [14]. Nevertheless, it has been reported that only 50-80% of patients with CDBDs have an APBJ [15] and only 19% patients with pancreaticobiliary reflux have an APBJ [10], suggesting that the APBJ theory cannot comprehensively explain the pathogenic mechanism of CDBDs.

It should be emphasized that pancreaticobiliary reflux and APBJ are 2 completely different concepts. Pancreaticobiliary reflux is the consequence of an abnormal confluent pancreaticobiliary juice, while abnormal anatomical changes result in an APBJ. However, these 2 concepts are confused and frequently considered interchangeable, leading to misunderstanding of the pathogenic mechanism of CDBDs.

Pancreaticobiliary reflux occurring in patients with a normal pancreaticobiliary junction is known as “occult pancreaticobiliary reflux” [16]. Sphincter of Oddi dysfunction (SOD) has been suggested to be the only possible explanation for occult pancreaticobiliary reflux [17–19]. Thus, we first proposed the theory that structural and functional abnormalities in the duodenal papilla include both an APBJ and a SOD dysfunction, both of which can lead to pancreaticobiliary reflux, eventually leading to CDBDs. Both APBJ and SOD dysfunctions are causes of adult CDBDs, with SOD dysfunction being more common [10]. This theory has been clinically confirmed [10] and provides a comprehensive explanation of the pathogenic mechanism of CDBDs.

### 2.3. Pathological Changes in CDBDs

Pathological changes of CDBDs include the morphological changes and histological changes. Cystic dilatation can occur at any segment of the extrahepatic or intrahepatic bile ducts and can be divided into 2 types of morphological changes: cystic dilatation and fusiform dilation [12]. The most common type is cystic dilation of the common bile duct. Different types of cystic dilatations exhibit varying extents of morphological changes [3, 12]. Determining the type of CDBDs preoperatively is crucial for choosing the appropriate surgical method.

During pancreaticobiliary reflux, the pancreatic juice erodes the entire biliary tree. Unlike a tumor, there is no clear histological boundary between a lesioned and normal bile duct. During surgery, it is difficult to identify the boundary between the lesioned and the normal bile duct by visual inspection or choledochoscopy. Thus, radical resection is performed based on morphological changes rather than histological changes.

According to the duration of the disease and the severity of the lesion, the dilated bile duct wall may present with different extents of histological changes. For those with a short duration or mild lesion, the histological changes are mild inflammation of the inner bile duct wall, and the bile duct mucosa shows degenerative changes. Nevertheless, the deep tissue of the bile duct wall has not been damaged, and its tissue structure is close to that of normal tissue. At this stage, the histological changes of the bile duct wall can be reversed after elimination of the cause; hence, the histological changes are reversible. In our practice, we have found that performing hepaticojejunostomy on the bile duct wall with reversible pathological changes can achieve proper bile duct flow and satisfactory outcomes [8].

As the disease progresses, the fibrosis of the bile duct wall is aggravated, and the wall of the capsule gradually thickens and consists of dense fibrotic inflammatory tissue with little smooth muscle. There is little or no epithelium covering the mucosa, and in some cases, there are ulcers and fibrous calcification. The blood supply to the bile duct wall is impaired. At this stage, even with elimination of the cause, the pathological changes and functions of the bile duct wall cannot be restored. The irreversible nonfunctional lesioned bile duct should be radically resected as much as possible during surgical treatment.

### 2.4. Mechanism of Malignant Transformation in CDBDs

Malignant transformation of CDBDs is not uncommon, and it is important to understand that the mechanisms of preoperative and postoperative malignant transformations are completely different. The pathogenic mechanism of the long-term erosion of refluxed pancreatic juice on the bile duct wall, combined with bacterial infection, further enhances malignant transformation at the lesioned bile duct wall [3, 20].

The mechanism of postoperative malignant transformation is because proper bile duct flow is not achieved by surgical treatment, leading to repeated postoperative reflux cholangitis and eventually malignant transformation within the cystic ducts [8]. Postoperative malignant transformation caused by incomplete resection is not because the remnant lesioned bile duct undergoes malignant transformation, but because the remnant lesioned bile duct forms fibrous scars, leading to bile duct stricture and biliary-enteric anastomotic stricture [8]. The stricture markedly impacts bile duct flow and leads to a series of postoperative complications, including malignant transformation.

### 2.5. Principles of Surgical Treatment for CDBDs

The general principles of surgical treatment for CDBDs should include elimination of the cause, excision of the lesions, establishment of proper bile duct flow, and management of secondary lesions.

#### 2.5.1. Elimination of the Etiology

Elimination of the etiology refers to preventing bile duct erosion from pancreatic juice due to elevated biliary pressure. This is done by establishing biliopancreatic diversion. The surgical procedure is to perform a Roux-en-Y hepaticojejunostomy to divert bile flow; this procedure has been clinically confirmed as the most practical and feasible surgical method for many years [2, 21].

#### 2.5.2. Excision of the Lesion

Excision of the lesioned bile duct is a crucial step of the whole surgical procedure for CDBDs. The irreversible nonfunctional lesioned bile duct should be radically resected as much as possible. The remnant lesioned bile duct may form fibrous scars at anastomotic stoma, and fibrous scar contraction will induce bile duct stricture and biliary-enteric anastomotic stricture, in turn affecting bile duct flow and causing a series of postoperative complications. Radical resection can effectively reduce the rate of postoperative complications and malignant transformation [2, 9, 11].

#### 2.5.3. Establishment of Proper Bile Duct Flow

Establishing proper intrahepatic bile and pancreatic secretion flow is the most important step. Our study has revealed that proper bile duct flow, rather than radical resection, is the most crucial factor determining the long-term postoperative outcomes in Todani type Ia, type Ic, and type IVa CDBDs [8]. Moreover, the absence of proper bile flow is a greater risk factor for poor long-term treatment effects for type Ia and type IVa patients, as compared with incomplete excision [8]. Establishing proper bile flow can ensure good long-term outcomes in complicated cases when complete excision is not feasible.

#### 2.5.4. Management of Secondary Lesions

The secondary lesions in patients with CDBDs include secondary bile duct stones, secondary intrahepatic infection, bile duct stricture due to long-term repeated infection, partial liver atrophy caused by long-term biliary obstruction, and portal hypertension secondary to biliary cirrhosis. Management of these secondary conditions during surgery can further ensure proper postoperative proper bile duct flow, improve postoperative liver function, and improve the long-term surgical outcomes.

### 2.6. Surgical Treatment for Type I CDBDs and Key Technical Notes

The current standard treatment for type I CDBDs is radical resection of cysts and reconstruction with an end-to-side Roux-en-Y hepaticojejunostomy [22, 23]. The general key points of surgical treatment for type I CDBDs are to radically resect the lesioned bile duct and to establish a proper bile duct flow. Surgical resection of the intrapancreatic and hilar lesioned bile duct is challenging.

#### 2.6.1. Radical Resection of the Lesioned Intrapancreatic Bile Duct

Management of the lesioned intrapancreatic bile duct is a common issue for type Ia and type Ic cases. It should be particularly emphasized that radical resection of the lesioned intrapancreatic bile duct is necessary during primary surgery. The presence of a remnant intrapancreatic cystic, dilated duct will lead to the formation of dead space within the pancreas where pancreatic juice may be forced into due to the APBJ [13, 24]. The reflux of the duodenal juice in the cyst, along with the erosion due to pancreatic enzymes, causes cholangitis in the cystic ducts [25]. Repeated infections induce further damage to the duodenal papilla, resulting in papillary stenosis or dysfunction, eventually leading to recurrent pancreatitis and cholangitis [25, 26].

Radical resection of lesioned intrapancreatic bile ducts is challenging, especially when the cystic lesion extends deeply into the pancreas [27]. We have developed a posterior pancreatic approach for radical resection of lesioned intrapancreatic bile ducts, which greatly improves the success rate of radical resection and surgical safety by reducing the incidence of intraoperative hemorrhage and injury to the pancreatic duct [11]. Briefly, the duodenal side of the peritoneum is opened, and the head of the pancreas and the duodenum are elevated and rotated to the left to expose the pancreas posterior to the pancreatic bile duct. After incision of the pancreatic tissue posterior to the bile duct, the cystic, dilated ducts are stripped along the adventitia, and the cyst is excised at 2 mm from the junction with the pancreatic duct, followed by closing the distal opening with suture. In addition to protecting the main pancreatic duct, it is essential to ensure the proper flow of the pancreatic juice after resection.

Both intraductal and periductal approaches are combined to strip the lesioned ducts with the assistance of a choledochoscope. Separating the lesioned ducts along the gap between the outer wall and the pancreatic tissue is relatively safe and caused less bleeding. In case of massive intraoperative hemorrhage, the posterior superior pancreaticoduodenal artery is ligated. In the case of severe inflammation around the bile duct, radical resection may not be achieved in order to avoid serious postoperative complications. However, it must be emphasized that the formation of a dead space should be strictly avoided during cyst removal, which can be accomplished by suturing and destruction of the bile duct mucosa.

#### 2.6.2. Management of a Hilar Lesioned Bile Duct

The key points to the resection of a lesioned hilar bile duct are to prevent postoperative biliary-enteric anastomotic stricture and hilar biliary stricture and to prevent stricture-induced severe postoperative complications, including recurrent reflux cholangitis, hepatolithiasis, biliary cirrhosis, liver abscess, and cholangiocarcinoma [28, 29]. Postoperative biliary-enteric anastomotic stricture is the primary reason for the high incidence of complications and malignancy [30–32].

For Todani type Ic cases, after surgical resection of the extrahepatic lesioned bile duct, the hilar bile duct can be used for the biliary-enteric anastomosis because the diameter of the hilar bile duct is large enough. This anastomosis can be performed well and will not lead to postoperative biliary-enteric anastomotic stricture and hilar biliary stricture.

**Figure 2:**
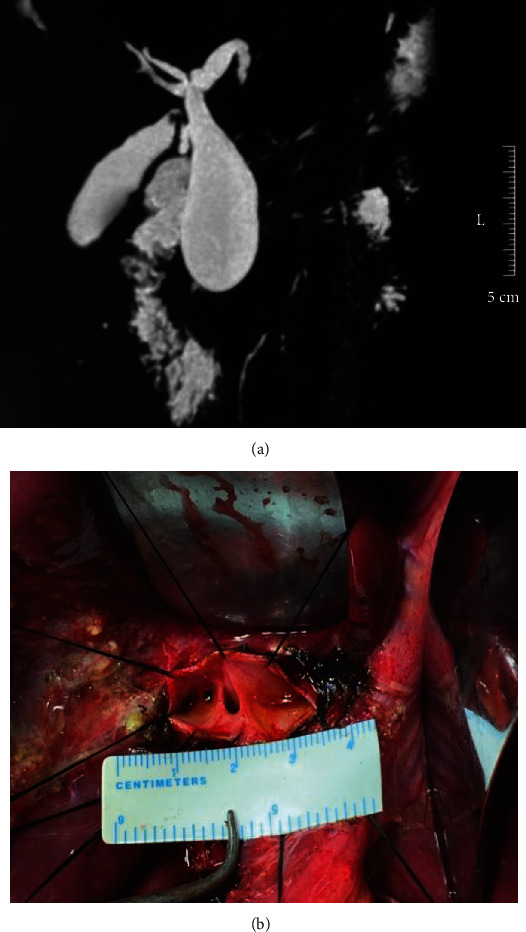
(a) Ultrasonographic image of type Ia CDBD. (b) Completed appearance of a hilar ductoplasty in type Ia CDBD.

For Todani type Ia cases (Figure 2(a)), after radical resection, due to the stenosis in the hilar bile duct, the diameter of the hilar bile duct is relatively small, causing certain difficulty with the subsequent biliojejunal anastomosis and a high incidence of postoperative biliary-enteric anastomotic strictures [6].

Hilar ductoplasty is used to enlarge the diameter of the hilar bile duct at the anastomotic opening (Figure 2(b)) and is typically performed to correct anastomotic stricture following a primary operation for CDBDs [9]. In 2005, we adopted hilar ductoplasty to enlarge the diameter of the hilar bile duct during the primary operation for type Ic cases, followed by the Roux-en-Y hepaticojejunostomy [6]. This method overcomes the problem of the small diameter of the hilar bile duct and significantly reduces the incidence of postoperative biliary-enteric anastomotic stricture and improves the overall prognosis in terms of biliary function [6]. During hilar ductoplasty, the size of the anastomotic opening of the hilar bile duct should be large enough to simultaneously ensure proper bile duct flow and prevent postoperative anastomotic stricture of the biliojejunal junction. Excessively dissecting the hilar hepatic duct is inappropriate as it can lead to severe postoperative complications and make subsequent surgical management difficult. In addition, the dissected area in the hilar hepatic duct should be limited to reduce operative trauma. We suggest that the optimal diameter is around 3.0 cm, ranging from 2.0 to 4.0 cm.

After hilar ductoplasty, the opening of the hilar bile duct is enlarged, and the orientation of the anastomosis between the hilar bile duct and the jejunum is similar to that between the common bile duct and the jejunum. Therefore, this surgical approach is a “side-to-side” Roux-en-Y hepaticojejunostomy. For side-to-side Roux-Y hepaticojejunostomy following hilar ductoplasty, the hilar bile duct and jejunum are anastomosed in a side-to-side manner, and the anastomotic junction is sutured with 4/0 or 5/0 absorbable sutures. The mode of anastomosis is determined based on the status of the bile ducts after hilar ductoplasty. It is recommended to use an intercostal anastomosis. If the anastomotic opening is large enough, continuous anastomosis at the posterior wall could be performed but with interrupted anastomosis at the anterior wall.

**Figure 3:**
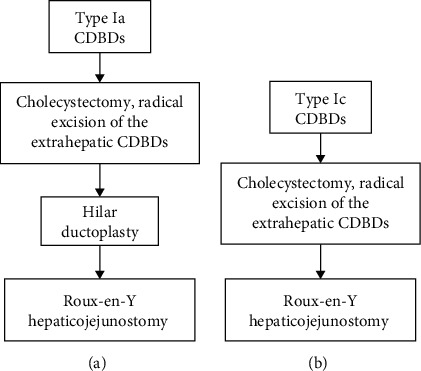
Flow chart of surgical treatment for type Ia (a) and type Ic CDBDs (b).

In summary, the standardized surgical procedure for type Ia CDBDs is radical excision of the extrahepatic CDBD, followed by the hilar ductoplasty and a side-to-side Roux-en-Y hepaticojejunostomy (Figure 3(a)), while that for type Ic CDBDs is radical excision of the extrahepatic CDBD, followed by Roux-en-Y hepaticojejunostomy (Figure 3(b)).

#### 2.6.3. Surgical Treatment for Type IVa CDBDs and Key Technical Notes

In the surgical treatment of type IVa cases, it is necessary to properly manage the intrahepatic dilated lesioned bile duct, in addition to the extrahepatic lesioned bile duct [7]. The intrahepatic dilated bile ducts can only be resected thoroughly through liver resection and extrahepatic cyst excision [33]. However, there are large variations in the morphology and distribution of intrahepatic lesioned bile ducts in type IVa cases. Therefore, surgical treatment for type IVa cases is the most difficult. Preoperative imaging including abdominal ultrasound (US), computed tomography (CT), magnetic resonance cholangiopancreatography (MRCP), and endoscopic retrograde cholangiopancreatography (ERCP) should be performed to adequately locate and qualitatively diagnose the lesioned dilated bile ducts and the involvement of bile ducts and blood vessels. If necessary, 3D imaging techniques can be used to facilitate formulating a surgical plan.

**Figure 4:**
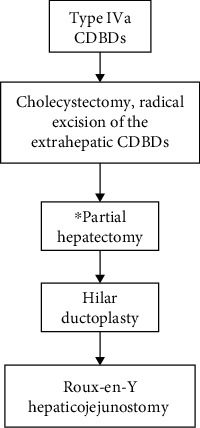
Flow chart of surgical treatment for type IVa CDBDs. ^∗^If the proper flow of intrahepatic bile duct can be achieved through hilar ductoplasty, partial hepatectomy can be omitted.

Despite the large morphological variations in type IVa cases, the surgical methods can be divided into 2 categories based on the need for combined partial hepatectomy [7]: (1) radical excision of the extrahepatic CDBDs, followed by hilar ductoplasty and Roux-en-Y hepaticojejunostomy and (2) radical excision of the extrahepatic CDBDs, followed by partial hepatectomy and Roux-en-Y hepaticojejunostomy. The flow chart of surgical treatment for type IVa CDBDs is shown in Figure 4.

The method of excision of extrahepatic lesioned ducts in type IV cases is the same as that in type I cases. Owing to the involvement of intrahepatic bile ducts in type IVa cases, radical resection requires combined partial hepatectomy. The treatment of intrahepatic biliary cysts needs to be evaluated according to the severity and extent of the lesioned bile ducts [7]. The scope of hepatectomy should not be arbitrarily expanded to achieve radical resection. Not all type IVa cases require combined partial hepatectomy. To establish proper bile flow in type IVa cases, the diameter of the outflow bile duct should be greater than the maximum diameter of the intrahepatic bile duct [7]. In practice, it is necessary to design an individualized surgical plan based on the severity and distribution of the intrahepatic lesioned bile ducts for each case. There are 4 surgical strategies for type IVa cases, based on the severity and distribution of intrahepatic lesioned bile ducts.

1. In cases with wide distribution, but relatively slight severity of intrahepatic lesioned bile ducts, hilar ductoplasty is sufficient to achieve a proper intrahepatic bile flow. The suggested surgical procedure is radical excision of the extrahepatic lesioned ducts, followed by hilar ductoplasty and Roux-en-Y hepaticojejunostomy
2. For cases in which the distribution of intrahepatic lesioned bile ducts is limited, and the intrahepatic lesions are confined to a segment or a lobe of the liver, morphological radical resection can be achieved by combined partial hepatectomy. The suggested surgical procedure is radical resection of extrahepatic lesioned ducts, followed partial hepatectomy and Roux-en-Y hepaticojejunostomy
3. In cases with a wide distribution and relatively high severity of intrahepatic lesioned bile ducts, the suggested surgical procedure is radical excision of the extrahepatic lesioned ducts, followed by partial hepatectomy and Roux-en-Y hepaticojejunostomy. Partial hepatectomy mainly includes left or right hepatic resection, but left or right hepatic trisegmentectomy is occasionally adopted
4. For cases with wide distribution and high severity of intrahepatic lesioned bile ducts, neither hilar ductoplasty nor partial hepatectomy can establish proper intrahepatic bile flow. If the patient does not have obvious clinical symptoms, surgery may be postponed with close clinical observation. If clinical symptoms are severed and bile duct infection is frequent, liver transplantation should be considered

## 3. Conclusion

Based on findings from clinical and pathological studies, a comprehensive theoretical system for the surgical treatment of CDBDs has been established, which corrects many previous theoretical misunderstandings. Using these principles, treatment outcomes for CDBDs can be improved.
